# Fetal craniofacial anomalies in amniotic band sequence

**DOI:** 10.1007/s00247-024-05993-7

**Published:** 2024-07-19

**Authors:** Aakanksha Sriwastwa, Beth M. Kline-Fath, Karin S. Bierbrauer, Charu Venkatesan, Usha D. Nagaraj

**Affiliations:** 1 Department of Radiology, University of Cincinnati Medical Center, 3188 Bellevue Avenue, Cincinnati, OH 45219 USA; 2 Department of Radiology, Cincinnati Children’s Hospital and Medical Center, 3333 Burnett Avenue, Cincinnati, OH 45229 USA; 3 Department of Neurosurgery, Cincinnati Children’s Hospital and Medical Center, 3333 Burnett Avenue, Cincinnati, OH 45229 USA; 4 Department of Neurosurgery, University of Cincinnati Medical Center, 3188 Bellevue Avenue, Cincinnati, OH 45219 USA; 5 Department of Neurology, Cincinnati Children’s Hospital and Medical Center, 3333 Burnett Avenue, Cincinnati, OH 45229 USA; 6 Department of Neurology, University of Cincinnati Medical Center, 3188 Bellevue Avenue, Cincinnati, OH 45219 USA

(Fig. 1)

**Fig. 1:**
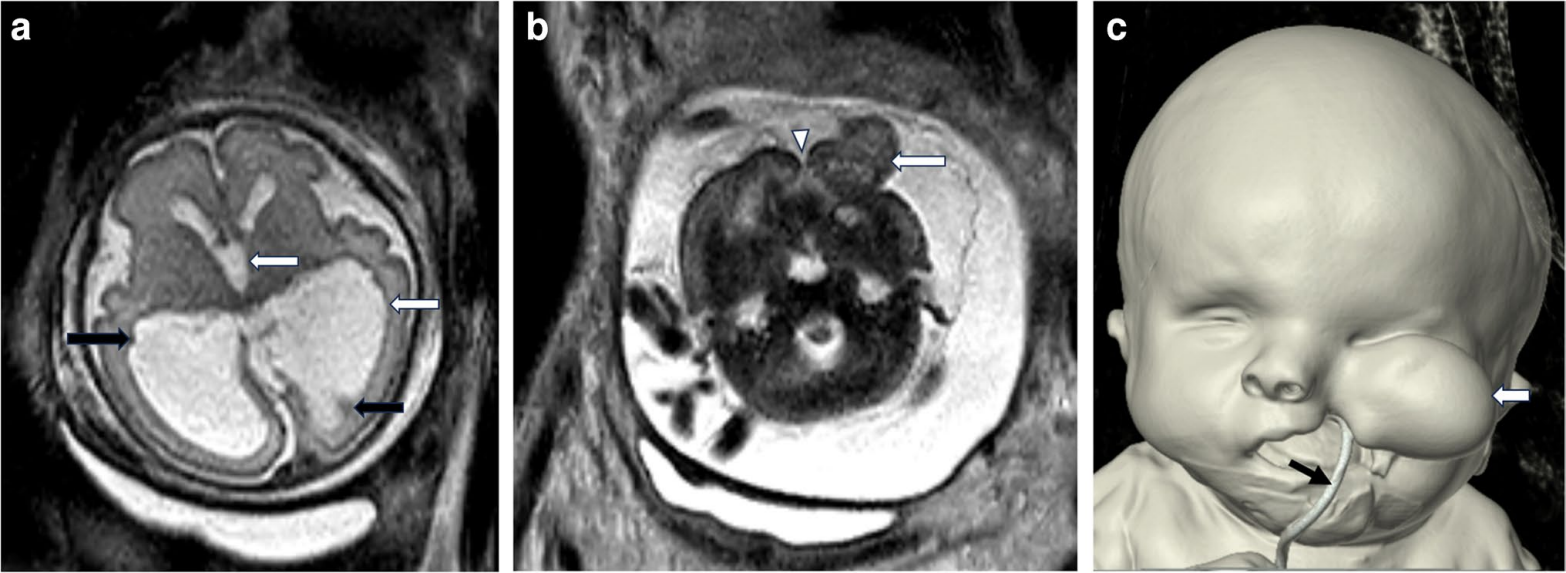
A 36-year-old gravid multipara woman was referred for a fetal magnetic resonance imaging (MRI) at 30-week gestational age after routine prenatal ultrasound revealed ventriculomegaly in one of the twins. **a** Axial T2-SSFSE image of the  fetal brain demonstrates ventriculomegaly involving bilateral lateral and third ventricles (white arrows), and multiple subependymal nodules (black arrows) consistent with periventricular gray matter heterotopia. **b** Axial T2-SSFSE image of the fetal face demonstrates left-sided cleft lip and cleft palate (arrowhead) and an extruding soft tissue lesion (arrow) arising from the left orbital/maxillary region with mixed T2 signal intensity. Postnatal MRI (not shown) confirmed these findings and additionally revealed a left closed lip schizencephaly. **c** Three-dimensional surface and volume-rendered reformatted computed tomography image demonstrates left paramedian cleft lip, palate and maxilla, and associated facial lesion (white arrow). Orogastric tube (black arrow), right microphthalmia, and nasal septal deviation are also noted

## Supplementary Information

Below is the link to the electronic supplementary material.

Supplementary file1 (MP4 346 kb)

## Data Availability

The data that support the findings of this study case are available from the corresponding author upon reasonable request. The data are not publicly available due to restrictions related to patient privacy, but can be made available upon request to personnels who meet the criteria for access to confidential data. The data are stored in a secure repository at the Cincinnati Children's Hospital and Medical Center.

